# Green Formation of Spherical and Dendritic Silver Nanostructures under Microwave Irradiation without Reducing Agent

**DOI:** 10.3390/ijms13078086

**Published:** 2012-06-28

**Authors:** Monir Noroozi, Azmi Zakaria, Mohd Maarof Moksin, Zaidan Abd Wahab, Alam Abedini

**Affiliations:** Physics Department, Universiti Putra Malaysia, 43400 UPM Serdang, Selangor D.E., Malaysia; E-Mails: monir.noroozi@gmail.com (M.N.); maarof@science.upm.edu.my (M.M.M.); zaidan@science.upm.edu.my (Z.A.W.); abedini_alam@yahoo.com (A.A.)

**Keywords:** microwave, silver nanostructures, silver nanoparticles

## Abstract

The rapid and green formation of spherical and dendritic silver nanostructures based on microwave irradiation time was investigated. Silver nanoparticles were successfully fabricated by reduction of Ag^+^ in a water medium and using polyvinylpyrrolidone (PVP) as the stabilizing agent and without the use of any other reducing agent, and were compared with those synthesized by conventional heating method. UV–vis absorption spectrometry, transmission electron microscopy (TEM), atomic absorption spectroscopy (AAS) and photon correlation spectroscopy (PCS) measurements, indicated that increasing the irradiation time enhanced the concentration of silver nanoparticles and slightly increased the particle size. There was a lack of large silver nanoparticles at a high concentration, but interestingly, the formation and growth of silver dendrite nanostructures appeared. Compared to conventional heating methods, the silver nanoparticle suspension produced by irradiated microwaves was more stable over a six-month period in aqueous solution without any signs of precipitation.

## 1. Introduction

The study of nanofluids with metal nanoparticles (NPs), particularly silver nanoparticles (AgNPs), is important in order to be utilized in different fields such as thick-film conductor conductivities, optical biosensors, biomedicine and medicine because of their antibacterial properties [1]. The high quality and nearly monodispersed NPs contribute to the size-dependent nanomedicine applications of AgNPs. In order to get dispersed silver nanoparticles in solvents, polyvinylpyrrolidone (PVP) is one of the commonly used agents that cover the AgNPs surface. The chain length of the surfactant polymer is protective and has been employed as a stabilizer and for the control of morphologies and sizes of Ag nanostructures [2]. Chemical reduction methods are usually used to synthesize AgNPs. Most chemical reducing reactions require elevated temperatures for a higher reaction rate. The energy used to heat up the media can be conventional heating [3], laser irradiation [4], ultrasound [5], UV irradiation [6], *etc.*

Generally, microwave (MW) technology is a viable avenue for the greener synthesis of nanomaterials [7–9] and metallic nanoparticles [10–13] because it is clean, rapid, and simple to use. Fast nucleation during the reaction leads to formation of high concentrations and more uniform size distributions [14]. However, MW irradiation should be more environmentally friendly and requires less energy than conventional methods. MW heating conditions allow not only the preparation of spherical nanoparticles within a few minutes but also the formation of dendrites. Depending on the MW irradiation time, it is possible to produce different shapes of Ag nanostructures. Well-defined Ag dendrites have been prepared by reduction of AgNO_3_ in N,N-dimethyformamide (DMF) in the presence of PVP under MW irradiation [15]. The synthesis of Ag NP using a number of chemical reducing agents including NaBH4, ethanol, ethylene glycol and DMF has been well demonstrated by several groups [3,11,16]. However, there are a few reports on the use of water solvents in the absence of any reducing agents. Metal colloids in the absence of any reducing agents are free of pollution and can be applied in biomedical system. Besides, the characterization of single dispersed or hydrodynamic agglomerates of nanoparticles is also necessary in order to understand the potential toxicity of NPs on biological systems to ensure that results are reproducible [17]. The rate of silver reduction by water is significantly higher due to its high dielectric values (a dielectric loss of 12 and a dielectric constant of 78). Dielectric heating raises the temperature of the total volume of the reactants by transforming energy selectively in microwave absorbing materials [18]. In particular, the silver nanoparticles can be promptly protected in the water by using a few minutes of MW heating technique.

With higher temperatures, an increasing growth rate of NPs and larger NPs were obtained. In addition, the reduction reaction was completely stopped because the silver reaction could not happen at high temperatures in the presence of water solvent due to its boiling point temperature. Therefore, this corresponds to the agglomeration of silver NPs, and hence controlling the particle growth process is not very easy in this method. However, the reaction rate increased with increasing irradiation time in the microwave system [19]. The removal of high temperatures in microwave heating is possible, since it allows for more reduction reactions of Ag nanoparticles. Moreover, the MW heating process can produce nanoparticles very quickly and with a very narrow distribution.

In this work, we proposed a green synthesis method by reducing AgNO_3_ with different MW reaction times at a moderate temperature for the preparation of silver nanostructures. Water was chosen as the environmentally benign solvent in the preparation. Secondly, PVP as an environmentally benign polymer was selected as a ligand as well as nontoxic reducing agents, which have been used as food additives [20]. Finally, microwave irradiation was used as the heating style. The influence of irradiation time on the particle size and distribution of AgNPs was characterized not only by using UV–vis spectra and transmission electron microscopy (TEM) images but also by photon correlation spectroscopy (PCS) which is a novel technique allowing for the simultaneous measurement of particle size and their agglomeration state in suspensions [21]. A conventional heating method was also employed to synthesize AgNPs for a comparison of room temperature with 60 °C.

## 2. Experimental Section

### 2.1. Materials and Methods

Firstly, AgNO_3_ (99.98%, 4.35 g/cm^3^) was used as the silver precursor, which was obtained from Merck (Darmstadt, Germany). PVP (K25, MW–29000, Aldrich Chemistry, 1.2 g/cm^3^) was used as a stabilizer for the fabrication of AgNPs. In a typical procedure, AgNO_3_ (0.3 g) and PVP (0.3 g) were dissolved separately in 25 mL of water and were stirred for 15 min. Then one was added to the other. The resultant solution was stirred for 10 min the resulting solution was cooled to room temperature. The corresponding solution was placed into a Pyrex glass cylinder container with a volume of 60 mL. A series of Ag solutions were prepared in a microwave oven (Panasonic, NN-K574MF, multi-mode, rated power 1100W, 2.45 GHz) at different irradiation times (20, 40, 60 and 90 s (W1–W4)), with a pause every 20 s to cool the reaction vessel. The temperature of the sample was controlled (~60 °C) to prevent intense boiling of the solvents as well as aggregation of metal NPs. After microwave irradiation, the mixture was allowed to cool to room temperature.

The temperature of a MW oven can reach 60 °C in 20 s. For the conventional heating method, a portion of a similar concentration and treatment of silver nitrate and PVP in water was heated on a hot plate from room temperature to 60 °C during about 20 min. This was done input a similar amount of heat energy as with the MW heating, but at a slower heating rate. After the reaction, the microwave power was turned off and the sample was cooled to room temperature.

### 2.2. Characterization Methods

The fabricated colloidal AgNPs were characterized by UV–vis spectroscopy (UV-1650 PC-Shimadzu), TEM (Hitachi H-7100) for observations of AgNPs in dry condition, aided by UTHSCSA Image Tool (version 3.0; Texas, USA) for software particle size distributions, and PCS (Nanophox Sympatec GmbH) for particle size characterization in aqueous solutions. Samples for PCS analysis were diluted and then placed in a clean vial for measurement, and each experiment had to be repeated four times. The scattered light from a laser beam exhibits a wavelength shift due to Brownian motion of the suspended particles and from it the particle size distribution can be obtained. An atomic absorption spectrometer (AAS-S Series; Thermo Scientific, San Jose, CA, USA) was used to measure the concentration of AgNPs in the solutions.

## 3. Results and Discussion

### 3.1. MW Irradiation

A microwave oven converts only part of its electrical input into microwave energy. The efficiency was determined by measuring the microwave output power, divided by the power consumption of the oven [22]. The output power absorbed by the sample is different from the nominal power given by the microwave instrument, and normally this effective power is determined by means of a calorimetric method [23]. The experimental results show that the maximum output power of existing microwave ovens is 680 W, which is 61.8% of the nominal value of 1100 W.

In this work, the color of the prepared samples at different MW-irradiation times at a moderate temperature of 60 °C gradually changed from colorless for AgNO_3_/water suspension (W0) to brown (W1, W2) and finally to dark brown (W3, W4) depending on the absorbation time which indicated the formation of AgNPs in the water suspension. It has been suggested that the color of colloidal Ag depends on the size and the shape of clusters as well as on the surrounding medium [24].

#### 3.1.1. TEM Images

**Figure 1:**
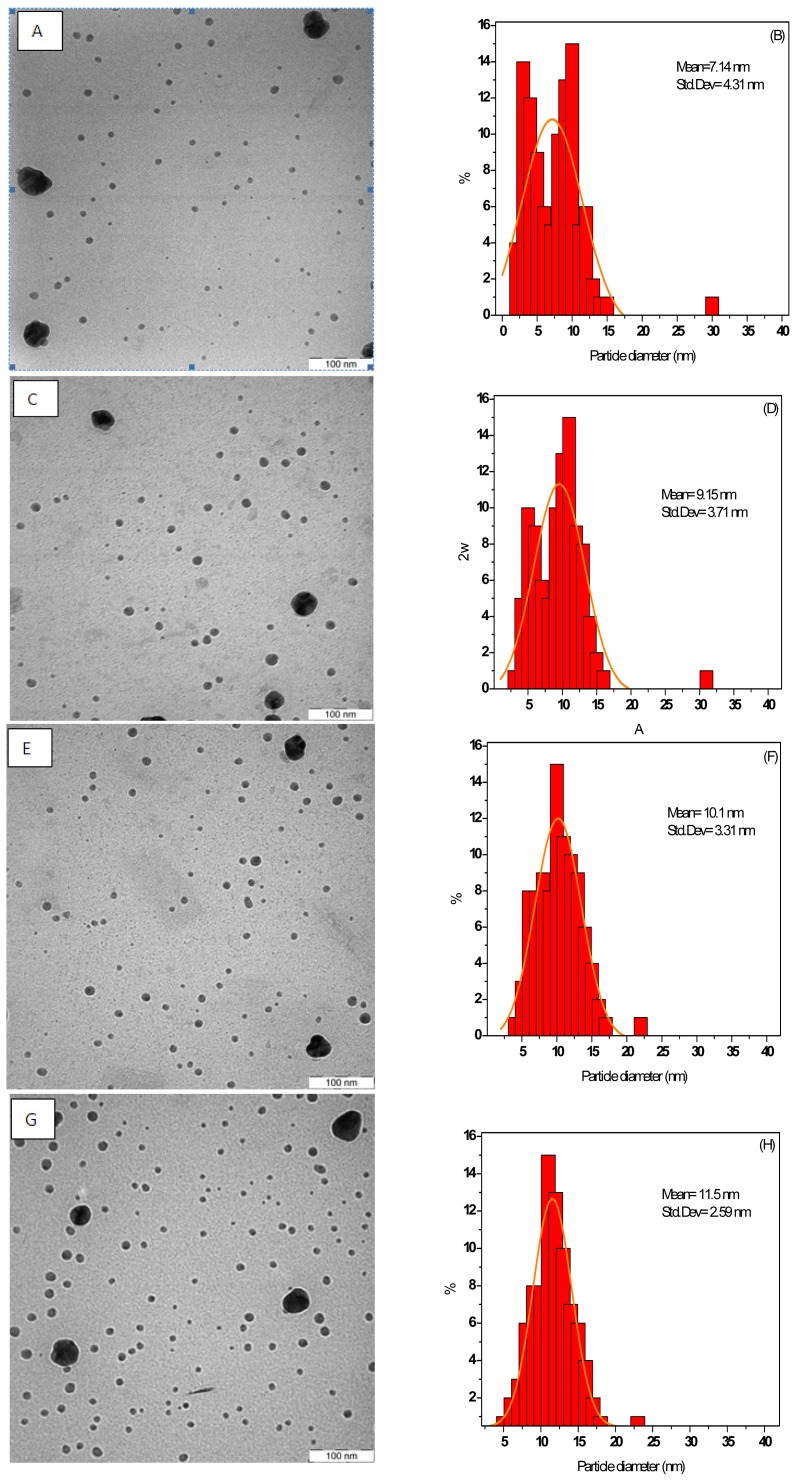
Transmission electron microscopy (TEM) images and the corresponding particle size distributions of Ag nanofluids at different MW-irradiation times: 20.s (**A**,**B**), 40.s (**C**,**D**), 60.s (**E**,**F**), and 90.s (**G**,**H**).

Figure 1 shows TEM images and their resultant size distributions of AgNPs in the colloidal solutions under MW irradiation periods. The mean diameter and the size distributions of AgNPs obtained by measuring the diameter and standard deviation of all particles in the images are shown beside each image.

**Figure 2:**
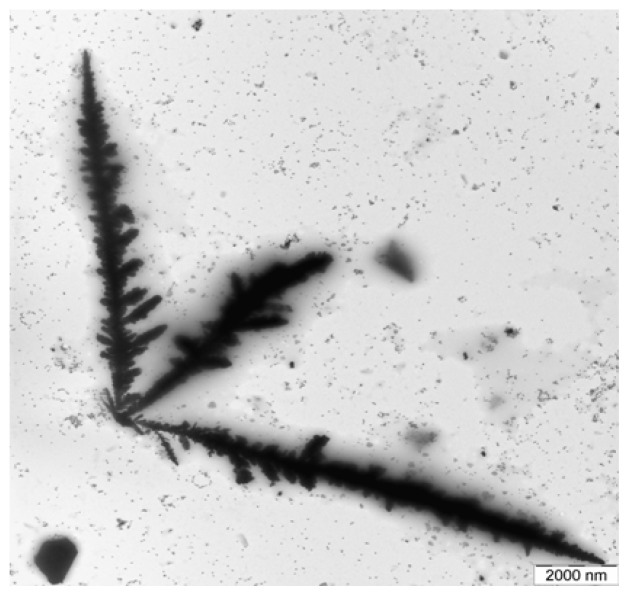
TEM micrographs of the dendritic nanostructures forming Ag trees.

As seen from Figure 1, the mean diameter of the AgNPs slightly increased and depended on MW irradiation times. In the AgNPs solution prepared at 20 and 40 s, AgNPs were formed with a broad size distribution and the mean particle size and standard deviation were about 7.14 ± 4.31 and 9.15 ± 3.71 nm (Figure 1 A,C), respectively. There is also evidence of small species as well as large ones, as can be seen from Figure 2 E and G. When the irradiation period was increased to 60 and 90 s, the mean particles size of AgNPs was increased considerably to 10.1 ± 3.31 and 11.5 ± 2.59 nm, respectively. This result also indicated that with increased MW-irradiation time, the AgNPs slightly increased in the solvent [14]. However, the result showed a narrow size distribution of the AgNPs, indicating that the particles were highly homogeneous after 90 s, when the MW-induced fragmentation of large AgNPs gradually commenced. On the other hand, there is an apparent increase in concentration and, probably, the amount of different species after irradiation. However, there is a lack of larger AgNPs, whereas many small NPs appeared. The particle size distribution obtained from the TEM image (Figure 1) shows that the combined effect of PVP thus helps in controlling the size of the particles.

Figure 2 shows a TEM image of AgNPs that was formed at 90 s of MW irradiation on the solution. As seen from Figure 2, the formation of dendritic nanostructures to the formation of silver trees was observed. When the concentration of the tne AgNPs precursor was increased, large dendritic nanostructures were formed [25]. Dendritic structures of AgNPs are formed at the interface when Ag ions were reduced in the presence of surfactants. Dendritic nanostructures of Ag have been produced by MW irradiation of AgNO_3_ in the presence of PVP [26]. According to the suggestion for the creation of Ag dendrites, MW power and PVP as a surfactant play important roles in the formation of Ag dendrites. Ag ions were reduced and metallic AgNPs were formed at the beginning of the reaction by MW irradiation of AgNO_3_ in the presence of PVP. Therefore, the interaction between Ag particles and MW energy can lead to the growth of the nanoparticles and an increase in temperature in nearby areas and may encourage new nuclei to appear at its boundary for further diffusion-limited growth. Consequently, the rate of the nucleation and development of the crystal control the creation of the dendrites [10].

#### 3.1.2. Photon Correlation Spectroscopy

**Figure 3:**
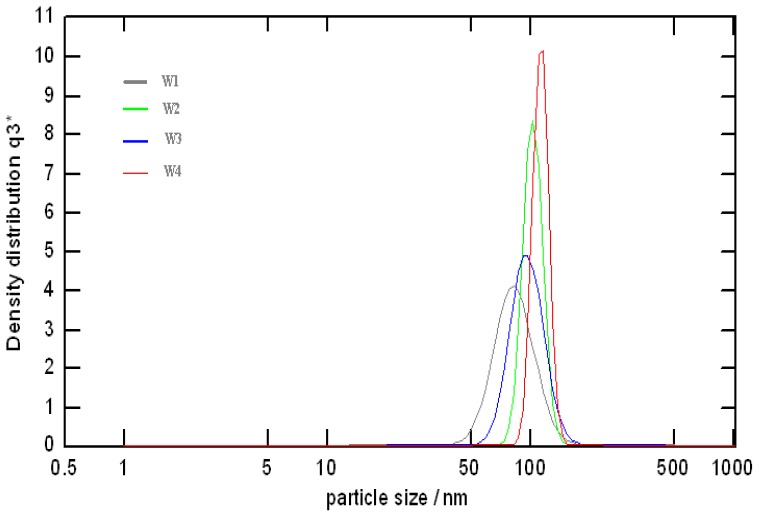
The hydrodynamic density size distribution of the colloidal Ag-NPs solutions that was measured by photon correlation spectroscopy (PCS) analyzer at different irradiation time: 20 (W1), 40 (W2), 60 (W3), and 90.s (W4).

Figure 3 shows the hydrodynamic density size distribution and the mean diameter of the colloidal AgNPs that were measured by PCS during MW irradiation periods. The mean diameters of AgNPs and the range of AgNPs particles in the solution are summarized in Table 1. The result showed that the maximum particle distribution and a monodispersion of particles were reached after 90 s of irradiation time, the distribution peaks being around 110.66 nm and the NPs sizes ranging from 99.14 to 123.65 nm, which was highly homogeneous. The sharper curve of distribution of the particle sizes is equivalent to a higher concentration of particle sizes in the nanofluid. Consequently, the present process can produce AgNPs steadily with almost equivalent quality and yield. This phenomenon was also confirmed in the particle size distribution obtained from the TEM image (Figure 1G). In all nanoparticle cases, the measured particle sizes were 10 times larger than the values obtained by TEM analysis of dried powder. The PCS measurement indicates the effective diameter of the agglomerates which implies that the surface of AgNPs was coated by PVP molecules, since they could not be detected in the solid state by TEM but were interpreted as a part of NPs in solution when recorded by PCS [12]. In the PCS measurement, all suspensions were identically diluted, and the differences in particle size and aggregate morphology reported here are valid for relative comparisons between the size distributions. However, the scattered light intensity is inversely proportional to the particle size. When the sample contains particles with a wide size distribution, or particles are mixed with surfactant coated molecules, the signal from larger particles can significantly block the signal from smaller particles, which makes accurate measurement difficult to realize. In this study, the large difference between the TEM images and the PCS data agrees with this assumption. Probably, the average size (~100 nm) of the PCS data (Figure 3) reflects the small amount of large AgNPs as shown in the TEM images (Figure 1).

#### 3.1.3. UV–vis Spectra

**Figure 4:**
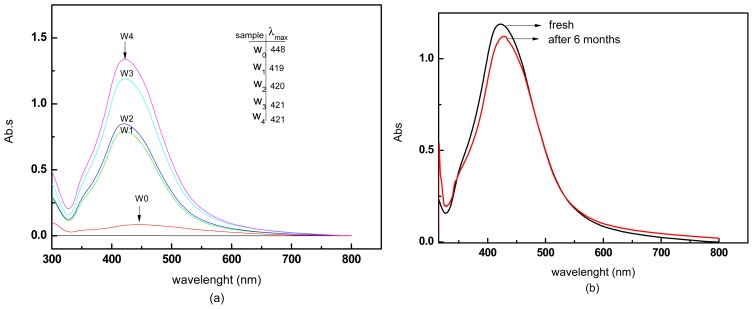
(**a**) Evolution of UV spectra during the formation of silver colloids in aqueous solution. Ag nanofluids at different MW irradiation times. The time of irradiation is shown on the traces. (**b**) The UV–vis spectra of AgNPs prepared using MW irradiation at 60.s (W3), fresh and after storage for six months at room temperature.

Figure 4(a) shows the absorption spectra of the colloidal AgNPs during different MW irradiation times. The UV–vis spectra showed symmetric peaks at about 420 nm, which is distinctive of surface plasmon resonance (SPR) features of AgNPs colloidal solutions, and a long tail lengthening toward the lower wavelength (UV range) [27]. However, the surface plasmon absorption band obtained from the AgNO_3_/water suspension (W0) before the MW-irradiation showed no sign of colloidal silver. Further a wide band absorption at around 450 nm is shown. The UV–vis spectra of the samples showed that the absorbance of AgNPs colloidal solutions increased sharply when the irradiation MW times were increased to 20, 40, 60, and 90 s, respectively, weherby the SPR peaks that appeared were slightly red-shifted and increased to 419 (W1), 420 (W2), 421(W3), and 421(W4) nm, respectively. The result showed that the maximum absorption and a narrow size distribution of the AgNPs were reached after 90 s of irradiation time, where it was highly homogeneous and the increase of absorbance was due to the surge of the number of NPs in the solution [28].

For a stability test of AgNPs, the absorption spectra of nanofluids of sample W3 was taken after storage for six months at room temperature (Figure 4b). The fact that the absorption peaks red shifted only slightly from 420 to 427 nm means that the agglomeration of AgNPs was quite small, and the small change in dispersion intensity shows that the AgNPs solution was quite stable. Therefore, AgNPs produced by MW irradiation produce stable AgNPs over a long period of time.

### 3.2. Formation of AgNPs with Conventional Heating

**Figure 5:**
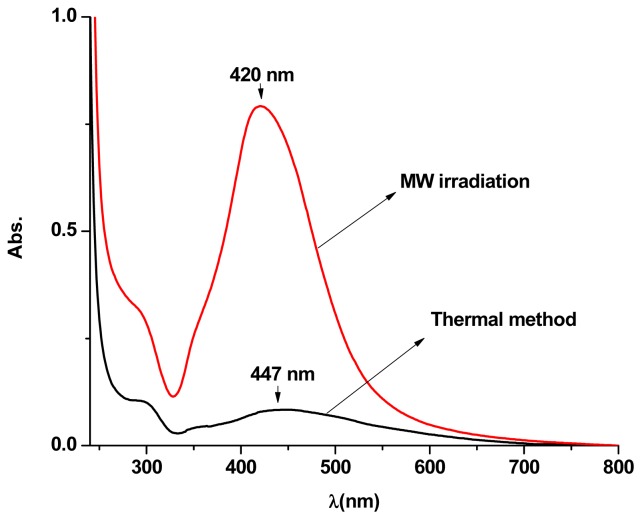
The UV–vis spectra of Ag-NPs prepared with MW irradiation and thermal method at the same temperature, the concentrations of silver nitrate and PVP are the same, while the temperatures reach 60 °C.

Figure 5 presents the UV–vis spectra of AgNPs dispersions synthesized using MW irradiation and conventional heating methods. The absorption peak intensity of the dispersion obtained by MW heating was higher and blue shifted (from 435 to 421 nm) compared to that of conventional heating. This blue shift and high peak indicate that the size of AgNPs prepared in MW heating is smaller and has a higher density [9] compared with that of conventional heating, which reflects more uniform size distributions. The obtained concentration of AgNPs by MW irradiation and conventional heating technique was 0.136 and 0.102 mg/L, respectively, confirming a high density of AgNPs produced by the MW method which confirmed the observation in the UV–vis spectra (Figure 5).

**Figure 6:**
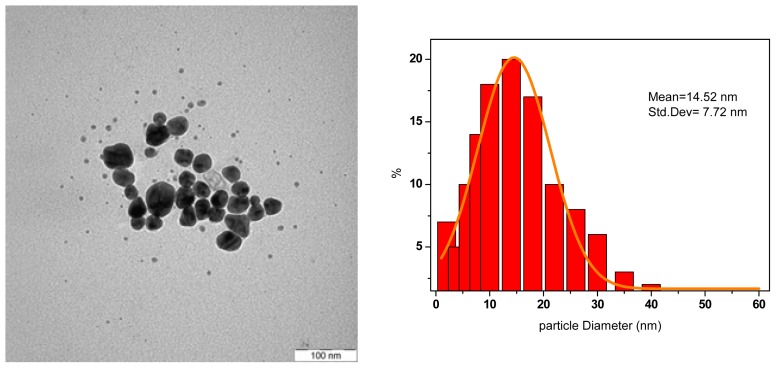
The TEM images of Ag-NPs synthesized by conventional heating method.

In Figure 6, the TEM image and the histogram clearly show that the AgNPs obtained from the conventional heating are bigger, 14.5 nm, than those produced with the MW heating method and not uniform in size, based on big standard deviation of 7.73 nm. The decrement in size of AgNPs produced by MW irradiation can be attributed to the efficiency of the electromagnetic field in heating the polarizing water molecules. With conventional heating, the solvent is heated by conduction and convection, so that there is a large temperature distribution within the solvent [10]. Rapid heating by MW irradiation produced a narrow size distribution of AgNPs because of homogeneous nucleation and subsequent crystal growth which was indicated by a superior yield of AgNPs [14].

## 4. Conclusions

The preparation of AgNPs in water and in presence of a PVP solution was done by MW irradiation as a simpler and greener method than heating by conventional method. The fabrication of narrowly dispersed silver nanoparticles was tuned in the size range of 7–17 nm in diameter with a standard deviation of σ ≤ 4.3–2.5 nm, by varying irradiation times only from 20 to 90 s. This showed that they were very stable in aqueous solution without any sign of precipitants over the long period of six months. The results indicated that by using MW irradiation, it is possible to obtain AgNPs of regular shape, narrow size distribution and a higher degree of crystallization in a reasonable synthesis time. The fast MW heating produces smaller and more uniform particle sizes and more density compared to that of the conventional heating method. Additionally, we believe that determination of the hydrodynamic diameter of the colloidal AgNPs under the PCS analyzer may be very useful for further nanomedicine applications to monitor their agglomeration state. Such highly-monodispersed and size-controlled AgNPs will contribute to the size-dependent nanomedicine applications of AgNPs.

## Figures and Tables

**Table 1:** The hydrodynamic mean diameter of Ag-NPs solutions prepared by MW irradiation in aqueous solutions at different irradiation times measured by PCS analyzer.

MW Irradiation Time (s)	Ag-NPs Mean Diameter (nm)	Standard Deviation (nm)
20	81.51	23.86
40	94.49	22.97
60	101.25	19.45
90	110.66	12.25
